# Bone Targeted Parathyroid Hormone Antagonists for Prevention of Breast Cancer Bone Metastases

**DOI:** 10.3390/cancers17172933

**Published:** 2025-09-08

**Authors:** Muralidharan Anbalagan, Tulasi Ponnapakkam, Binghao Zou, Jarvis Williams, Fouad Saeg, Matthew E. Burow, Robert C. Gensure, Brian G. Rowan

**Affiliations:** 1 Department of Structural and Cellular Biology, Tulane University School of Medicine, New Orleans, LA 70112, USA; manbalag@tulane.edu (M.A.); tponnapakkam@gmail.com (T.P.); bzou1@tulane.edu (B.Z.); jwilliams15@tulane.edu (J.W.); fsaeg@tulane.edu (F.S.); 2 Department of Chemistry, Xavier University of Louisiana, New Orleans, LA 70125, USA; 3 Section of Hematology & Medical Oncology, Department of Medicine, Tulane University School of Medicine, New Orleans, LA 70112, USA; mburow@tulane.edu; 4 Dartmouth Health Children’s, Geisel School of Medicine, Lebanon, NH 03756, USA; robert.c.gensure@hitchcock.org

**Keywords:** bone metastasis, metastatic breast cancer, parathyroid hormone antagonists, targeted therapy

## Abstract

Background/Objectives: Advanced breast cancer patients may develop bone metastases, leading to severe pain, fractures, and mortality. Current treatments have limited efficacy, necessitating targeted therapy approaches. Bone metastatic breast cancer cells secrete PTHrP that promotes tumor growth and bone degradation. Previous PTH/PTHrP antagonists failed clinically due to short half-life and insufficient bone targeting. The present study evaluated the following two novel bone-targeted PTH antagonists fused to a collagen-binding domain (CBD) for enhanced bone localization: PTH(7-33)-CBD and [W2]PTH(1-33)-CBD. Methods: Intra-tibial and intra-iliac breast tumor models in mice were used to evaluate drug efficacy in reducing tumor burden and bone destruction. Results: Bioluminescent imaging, X-ray, and micro-CT analysis revealed that both drugs significantly reduced tumor burden and osteolysis compared to control, with [W2]PTH(1-33)-CBD additionally improving trabecular bone structure. Drug efficacy was confirmed in both intra-tibial and intra-iliac breast tumor models. Conclusions: These findings identify CBD-fused PTH/PTHrP antagonists as a promising therapeutic strategy for breast cancer bone metastases.

## Simple Summary

Breast cancer frequently spreads to the bones, leading to pain, fractures, and other bone-related complications. Current treatments slow the disease progression but rarely halt it completely and may cause significant side effects. Parathyroid hormone-related protein (PTHrP) is a major driver of bone damage in this setting, stimulating bone breakdown and creating conditions that favor tumor growth. This study evaluated two bone-targeted drugs, PTH(7-33)-CBD and [W2]PTH(1-33)-CBD, that block the action of PTHrP. Each drug contains a collagen-binding domain that directs it to type 1 collagen in bone, concentrating the drug at the sites. Using two mouse models of breast cancer bone metastasis, a single injection of either drug substantially reduced tumor growth and prevented bone destruction. By delivering treatment directly to the bone, these PTHrP antagonists may offer a safer, more effective approach for managing skeletal metastases, with the potential to improve quality of life for patients.

## 1. Introduction

Bone is a common site of breast cancer metastasis, providing a favorable microenvironment for tumor growth. Nearly 40% of patients with bone metastases experience severe skeletal complications, including fractures, intractable pain, spinal cord compression, and neurological impairment, all of which contribute to increased mortality [1], tumor cells in bone become resistant to chemotherapy and radiation and may serve as a reservoir for further metastatic spread, organ failure, and eventual mortality.

Breast cancer cells that metastasize to bone secrete parathyroid hormone-related peptide (PTHrP), which activates PTH/PTHrP receptors (PTHR1) on osteoblasts, leading to the release of receptor activator of nuclear factor-kappaB (RANK) ligand [2,3,4]. RANK ligand induces osteoclast maturation, resulting in bone resorption, release of tumor-promoting factors, and expansion of metastatic lesions. PTHrP further enhances tumor growth through autocrine stimulation, perpetuating this cycle [5,6]. Previous attempts to block PTH/PTHrP activity using antibodies have been clinically ineffective, possibly due to inadequate bone targeting and off-target effects [7], particularly in the kidney. Current treatments, including bisphosphonates and denosumab, target osteoclast-driven bone resorption but have limited clinical efficacy, highlighting the need for novel therapeutic strategies [8,9].

PTHrP peptide antagonists block PTHR1 on osteoblasts and cancer cells, thereby inhibiting osteoclast activation and tumor progression. However, PTHrP antagonists have failed clinically due to short half-life, poor bone targeting, and adverse side effects [10,11]. To overcome these limitations, PTHrP antagonists were engineered with amino-terminal modifications to increase half-life. PTH(7-33)-CBD is a N-terminally truncated PTH antagonist (amino acids 7–33) fused to the collagen-binding domain (CBD) of the bacterial collagenase ColG from *Clostridium histolyticum*. [W2]PTH(1-33)-CBD retains the full-length PTH(1–33) sequence with a tryptophan substitution at position two and is also fused to the CBD. The PTH/PTHrp antagonist design was performed using mutational analyses by Shimizu et al. [12] where para-benzoyl-I-phenylalanine at position 2, Bpa2-PTH(1-34), was identified as the most potent antagonist. Consistent with these findings, our earlier work showed that substituting Bpa with a naturally occurring amino acid, tryptophan (W), resulted in compound W2-PTH(1-33)-CBD that exhibited markedly more antagonist activity than the parent comparator PTH(7-34), providing a strong rationale for its use in the current study [13]. Targeted delivery via the CBD is expected to concentrate PTHrP antagonists in the bone after a single subcutaneous injection at levels sufficient to counteract the action of tumor cell-derived PTHrP. By directly blocking PTHrP action in bone, these novel targeted antagonists may prevent the outgrowth of breast cancer bone metastases, thereby inhibiting bone metastatic tumor cells from seeding metastases in other tissues and ultimately reducing the risk of organ failure and patient death.

A previous study from this laboratory demonstrated that the fusion of CBD to the PTH-(7–33) and [W2]PTH(1–33) peptides retained the antagonistic properties of the parent antagonist and induced PTHR1-dependent apoptosis of MDA-MB-231 breast cancer cells in vitro [13]. More importantly, both agents significantly reduced tumor burden and osteolytic destruction in a preclinical bone metastasis model. The present study evaluates the therapeutic efficacy of PTH(7–33)-CBD and [W2]PTH(1–33)-CBD in mouse models of bone metastatic breast cancer.

## 2. Materials and Methods

### 2.1. Cell Culture

Bone-morphic (BM) MDA-MB-231-BM/luc+ cells were a gift from Yibin Kang at Princeton University [14]. MDA-MB-231-BM/luc+ cells were cultured in DMEM (Thermo Fisher Scientific, Waltham, MA, USA) supplemented with 10% fetal bovine serum (Gemini, Sacramento, CA, USA) and 1% penicillin/streptomycin (Invitrogen, Thermo Fisher Scientific, Waltham, MA, USA). Cells were maintained in a humidified environment of 5% CO_2_ at 37 °C.

### 2.2. PTH(7-33)-CBD and W2PTH(1-33)-CBD

PTH antagonist fusion peptides were synthesized by recombinant DNA techniques and kindly provided by BiologicsMD, Inc. (Boston, MA, USA). The bone-targeted PTHrP antagonists, PTH(7-33)-CBD and [W2]PTH(1-33)-CBD, were engineered by fusing a type I collagen–binding domain (CBD) to the C-terminus of the antagonist peptide sequence. This biologically inert CBD is 17 kDa; CBD was connected via a flexible GINS linker designed to provide spatial separation between the antagonist peptide and the targeting domain. This configuration was selected to preserve receptor-binding functionality of the antagonist while minimizing steric hindrance from the CBD. The W2 modification in [W2]PTH(1-33)-CBD was incorporated based on prior mutational analysis of PTH analogs, which identified Trp2 substitution as an optimal natural amino acid change to enhance antagonist activity.

### 2.3. Animal Experiments Using Intra-Tibial Injection of Breast Cancer Cells

Female Balb/c nude mice (6–8 weeks old) were purchased from Charles River Laboratories and maintained under pathogen-free conditions. The mice were divided into two groups of four mice per cage. Mice were then acclimated for 2 weeks in the vivarium and exposed to a 12/12 h light/dark period. The mice were given access to tap water and a diet of 18 percent protein, purchased from Harlan Company (Barton, IL, USA and Madison, WI, USA). Mouse experiments were performed according to the guidelines for the care and use of laboratory animals and were approved under Institutional Animal Care and Use Committee protocol #2941R4 from Tulane University, New Orleans. 2.5 × 10^5^ cells of a bone metastatic variant of MDA-MB-231 breast cancer cells expressing luciferase (MDA-MB-231-BM/luc+) were injected into the tibia marrow of nude mice [15]. MDA-MB-231-BM/luc+ cells have a high rate of osteotropism and very little metastasis to the lungs, thus permitting the evaluation of drug efficacy against metastatic burden in bone before the animals succumb to other systemic metastases [14]. Female nude mice received a single, subcutaneous (SC) injection (1000 µg/kg) of PTH(7–34), PTH(7–33)-CBD, [W2]PTH(1–33)-CBD or vehicle buffer 24 h before tumor cell injection into the tibia. A second drug dose was administered 4 weeks after tumor cell injection. The 1000 µg/kg dose was chosen as an effective dose based upon preliminary data and a prior studies that compared doses from (350 to 2000 µg/kg) [13]. Mice were weighed, imaged for tumor bioluminescence, and subjected to X-ray imaging weekly. At the end of the experiment (10 weeks), animals were sacrificed, blood was obtained, and bone tissue was prepared for histological and ex vivo micro-CT analysis.

### 2.4. Animal Experiments Using Intra-Iliac Artery Injection of Breast Cancer Cells

Intra-iliac artery injection of breast cancer cells into Balb/c mice was performed as previously described [16,17]. Briefly, Balb/c nude mice were anesthetized and restrained on a board. An incision was made on the skin along the line between the femoral and ilium bones. Muscles were separated gently to expose the common iliac artery. About 250,000 MDA-MD-231Luc+ cells/10 microliters were injected using a 36 g NanoFil needle. At the injection site, a cotton tip was used to press until the bleeding stopped (in about 3–5 min). The incisions were then sutured, and mice were monitored frequently until they were awake and moving normally. Animals that did not form measurable tumors were excluded from analyses. This experiment used [W2]PTH(1-33)-CBD, which showed the most significant overall drug efficacy in vitro and in vivo. [W2]PTH(1-33)-CBD was administered 24 h prior to tumor cell inoculation to one group of mice (days 0 and 40) and following tumor establishment in bone to another group of mice (days 14 and 44), allowing evaluation of both early and established disease settings.

### 2.5. In Vivo Bioluminescence and X-Ray Imaging

As previously described, an in vivo small animal imaging system (IVIS, PerkinElmer, Shelton, CT, USA) was used to measure metastatic growth in bone [18]. Briefly, mice were administered the substrate D-luciferin by intraperitoneal injection at 150 mg/kg in Dulbecco’s PBS (Invitrogen) and anesthetized (1–3% isoflurane). Ten minutes after substrate administration, the mice were placed into the IVIS imager, containing a light-tight camera, with continuous exposure to 1–2% isoflurane. Bioluminescence and X-ray images were acquired with Living Image software v4.8.2 (PerkinElmer, Shelton, CT, USA), and bone metastatic tumor growth was measured by marking the regions of interest (ROIs) from displayed images around the tumor sites and quantified as total photon counts or photons/sec in Living Image software. X-ray images obtained from the IVIS imager were analyzed using ImageJ software (NIH). Osteolytic destruction (defined as the region of reduced bone density) was determined and outlined, and the outlined area was measured in mm^2^.

### 2.6. Micro-Computed X-Ray Tomography

Mouse bones were removed from storage in alcohol and dried superficially on paper tissue, then wrapped in Parafilm to prevent drying during scanning and associated movement artifacts. Each plastic-wrapped bone was placed in a plastic/polystyrene foam tube, which was mounted vertically in a Skyscan 1172 scanner (Bruker, Billerica, MA, USA) sample chamber, for micro-CT imaging (50 kV, 200 μA, 4.3 μm voxel size, 1180 ms exposure time, 180° rotation, 0.6° rotation step, frame averaging ×2, and 0.5 mm Al filter). Reconstruction was performed with a modified Feldkamp algorithm in Skyscan Nrecon software(Micro Photonics Inc. Allentown, PA, USA), which facilitates network-distributed reconstruction on a personal computer. The time for reconstruction of a scan dataset is usually much shorter than the scan duration.

*a*. Selection of ROIs for trabecular and cortical bone

Both trabecular (metaphyseal) and cortical (metaphyseal-diaphyseal) bones were selected with reference to the growth plate. A cross-sectional slice was selected as a growth plate reference slice by moving slice by slice from the epiphysis through the growth plate and selecting the cross-sectional level at which the last remaining “bridge” of fine bony structures of primary spongiosa was broken. The reference slice could be identified in all micro-CT scans of the proximal tibia. The ROIs were selected with reference to the growth plate reference slice, as described above. Trabecular regions were defined as positions along the long axis of the tibia relative to the growth plate reference. The trabecular region commenced approximately 50 image slices from the growth plate level in the direction of the metaphysis and extended from this position for another 450 image slices. Within the trabecular region, the trabecular from cortical bone was separated with a freehand drawing tool for delineating complex ROIs. The selected cortical region comprised 200 image slices from the growth plate level in the direction of the metaphysis and extended from this position for 200 image slices. This site had relatively few remaining thin trabecular structures, in contrast to the much higher relative volume and number density of trabecular structures observed at the metaphysis close to the growth plate.

*b*. Morphometric analysis

The 3D morphometric parameters were calculated for the trabecular selected ROIs. Single gray threshold values were applied for the trabecular structures and ROI. Because the signal-to-noise ratios of the reconstructed images were high, further image processing steps on the binarized images, such as despeckle, were unlikely to improve measurement precision significantly. The 3D parameters were based on analyzing a marching cubes-type model with a rendered surface.

### 2.7. Statistical Analysis

Statistical comparisons between groups were performed using the Students’ *t*-test, and *p* < 0.05 was considered statistically significant. Statistical analyses were performed in GraphPad Prism 6.

## 3. Results

### 3.1. Drug Efficacy In Vivo Direct, Intra-Tibial Inoculation of Tumor Cells

**Figure 1:**
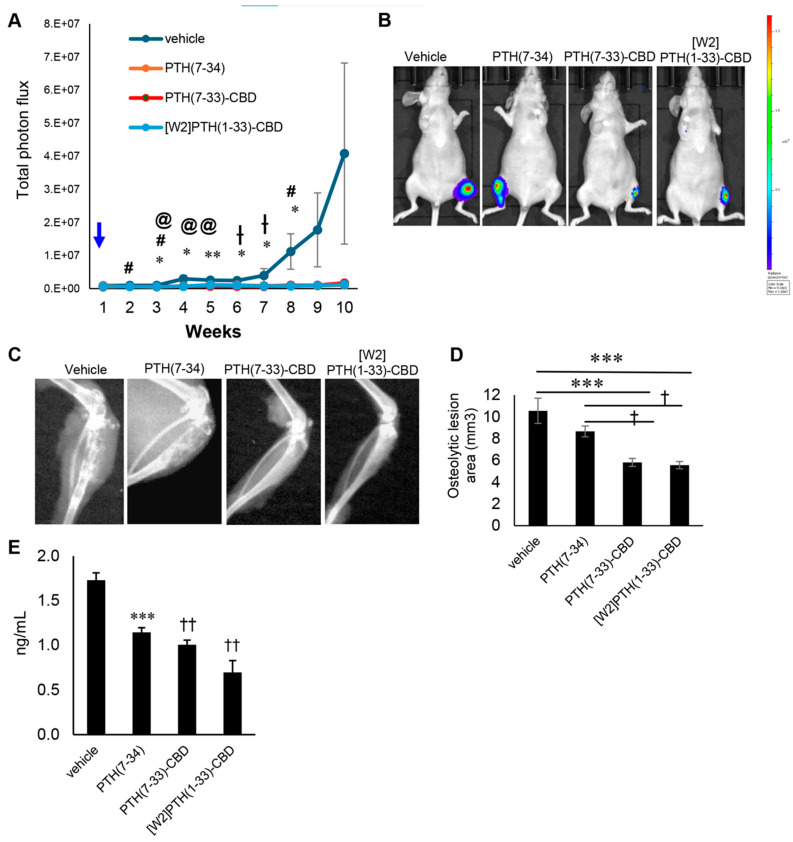
**PTH (7-33)-CBD inhibits bone metastatic MDA-MB-231 tumor growth in mice tibia.** MDA-MB -231-BM/Luc+ cells (2.5 × 10^5^ cells) were injected into the tibia marrow of nude mice. Animals received Vehicle, PTH(7-34) control drug, PTH(7-33)-CBD, and [W2]PTH(1-33)-CBD were given subcutaneously one dose of 1000 µg/kg once (n = 8). The molar concentration of PTH(7-34)was 4 times higher than PTH(7-33)-CBD and [W2]PTH(1-33)-CBD. (**A**) Tumor growth was quantitated by tumor bioluminescence using IVIS small animal imaging system and shown from week 1 to week 10, * denotes *p* < 0.05, ** *p* < 0.01 PTH(7-33)-CBD statistically significant compared to the vehicle; ^†^ denotes *p* < 0.05 statistically significant compared to PTH(7-34) control; # denotes *p* < 0.05 [W2]PTH(1-33)-CBD statistically significant compared to vehicle @ denotes *p* < 0.05 PTH(7-34) control statistically significant compared to vehicle. (**B**) Representative in vivo bioluminescence images of mice treated with vehicle, PTH7-34 control drug, PTH(7–33)-CBD and [W2]PTH(1-33)-CBD bearing intratibial breast metastatic tumor growth on week 10 after drug administration. Bioluminescent signal intensity (BLSI) is depicted using a color scale (right), where blue indicates low and red indicates high radiance levels (photons/sec/cm^2^/sr). (**C**) Representative Radiographic images of the proximal tibia from mice treated with vehicle, PTH(7-34) control, PTH (7-33)-CBD, and [W2]PTH(1-33)-CBD that had breast cancer bone metastases. MDA-MB-231 BM luc+ (2 × 10^5^ cells injected intra-tibially) 10 weeks after drug administration. (**D**) Quantitation of osteolytic lesion area using image J software in X-ray images of the proximal tibia from mice treated with vehicle, PTH(7-33) control, PTH(7–33)-CBD, and [W2]PTH(1-33)-CBD that had breast cancer bone metastases, 10 weeks post-drug administration. *** *p* < 0.001 compared to vehicle and ^†^ *p* < 0.05 compared to PTH(7-34) control. (**E**) Using ELISA, the bone resorption marker tartrate-resistant acid phosphatase (TRAP) levels were measured in mice serum 10 weeks after drug administration. *** *p* < 0.001 compared to vehicle and ^††^ *p* < 0.01 compared to PTH(7-34) control.

PTH(7-33)-CBD significantly reduced tumor burden in tibial bone from weeks 3–8post-tumor cell inoculation compared to vehicle (*p* < 0.05). [W2]PTH(1-33)-CBD, to a lesser extent, reduced tumor burden in bone on weeks 2, 3, and 8 (Figure 1A,B). PTH(7-34), a commercially available antagonist peptide lacking the CBD, required a four-fold higher concentration of PTH antagonists (with CBD) to significantly reduce tumor burden in bone at weeks 3–5 (Figure 1A,B). PTH(7-33)-CBD and [W2]PTH(1-33)-CBD, but not PTH(7-34), significantly attenuated osteolytic destruction of bones compared to vehicle (Figure 1C,D). Tartrate-resistant acid phosphatase (TRAP) was measured in serum collected from mice at the end of the experiment (week 10). TRAP is released by osteoclasts that mediate bone resorption. TRAP levels were significantly lower in PTH(7-33)-CBD, [W2]PTH(1-33)-CBD, and PTH(7-34) groups compared to vehicle (Figure 1E). These findings demonstrate that [W2]PTH(1-33)-CBD and PTH(7-33)-CBD reduce both bone metastatic tumor burden and osteolytic lesions. PTH(7-34) without the CBD fusion administered at a four times higher molar concentration could not prevent tumor-induced osteolytic bone destruction (Figure 1C,D).

**Figure 2:**
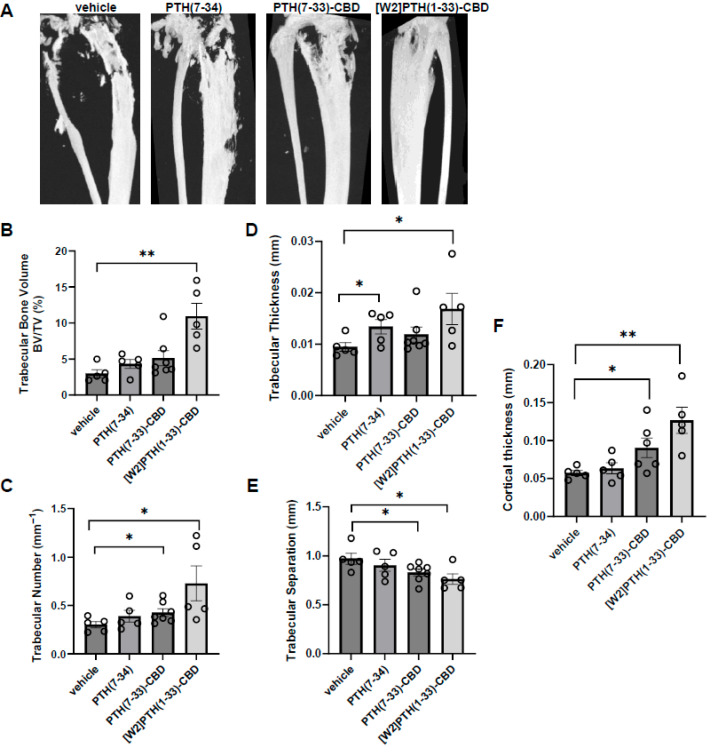
**PTH antagonists targeting bone reduce bone loss induced by breast cancer cells.** (**A**), Representative micro-CT images of tibia 10 weeks post-administration using a Bruker SkyScan 1172 ex vivo micro-CT scanner (n = 4–7/group). Proximal tibial bone architectural analysis by micro-CT scan and quantification (**B**), trabecular bone volume fraction (**C**), trabecular number (**D**), trabecular thickness (**E**), trabecular separation (**F**) cortical bone thickness (n = 4–7/group). * *p* < 0.05; ** *p* < 0.01 compared to vehicle.

Trabecular bone: [W2]PTH(1-33)-CBD significantly improved trabecular bone parameters compared to other groups. The [W2]PTH(1-33)-CBD group exhibited a trabecular bone volume fraction (BV/TV) more than twice that of the vehicle, PTH(7-34), and PTH(7-33)-CBD groups (*p* < 0.01) (Figure 2A,B). This increase was associated with a higher trabecular number that was more than double that of the vehicle group (Figure 2C). Additionally, trabecular thickness was significantly greater in the [W2]PTH(1-33)-CBD and PTH(7-34) groups (Figure 2D), while trabecular separation was reduced in both the PTH(7-33)-CBD and [W2]PTH(1-33)-CBD groups (Figure 2E). These findings indicated that [W2]PTH(1-33)-CBD effectively enhanced trabecular bone structure, potentially contributing to its therapeutic benefits in bone metastatic breast cancer.

Cortical bone: The [W2]PTH(1-33)-CBD and PTH(7-33)-CBD groups, but not the PTH(7-34) group, exhibited significantly greater cortical thickness than the vehicle group (Figure 2F). Taken together with the findings from trabecular bone analyses, these results indicated that [W2]PTH(1-33)-CBD, and to a lesser degree PTH(7-33)-CBD, significantly decreased both the trabecular and cortical bone destruction in tumor-bearing mice.

### 3.2. Histological Analysis of Intra-Tibial Tumors and Bone

**Figure 3:**
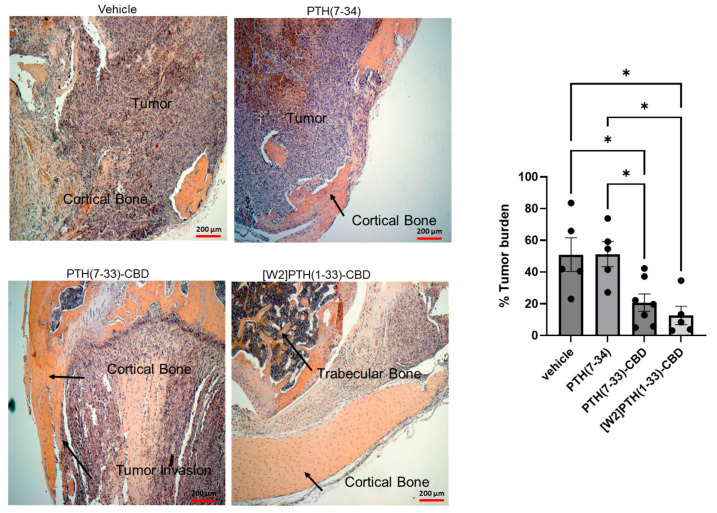
**Histological examination of mice tibia-treated PTH Antagonists**-representative H&E images of breast tumor in the tibia marrow and the surrounding space. Mice tibia were inoculated with 2.5 × 10^5^ MDA-MB-231-BM/luc+ cells. After euthanasia, the tibia was removed, fixed, decalcified, sectioned, and stained with H&E representative 5 × images for each treatment group are shown. Cortical bone, trabecular bone, and tumor in the bone were labeled. The bar graph shows the percentage tumor burden in bone measured using ImageJ software. * *p* < 0.05.

In the vehicle group, most bony tissue was replaced by tumor (Figure 3, panel 1), whereas in the [W2]PTH(1-33)-CBD group, preservation of both cortical and trabecular bone was observed (Figure 3, panel 2). The PTH(7-33)-CBD group exhibited moderate tumor invasion into the cortical bone and limited preservation of the trabecular bone (Figure 3, panel 3). The diaphysis near the proximal tibial growth plate was visible only in animals administered the antagonist compounds but was absent in the vehicle and PTH(7-34) groups (Figure 3, panels 2, 3, and 4). In the PTH(7-34) group, images were taken near the expected diaphyseal region where most bone tissue was visible (Figure 3, panel 4)

### 3.3. Drug Efficacy In Vivo Intra-Iliac Artery Inoculation of Tumor Cells

**Figure 4:**
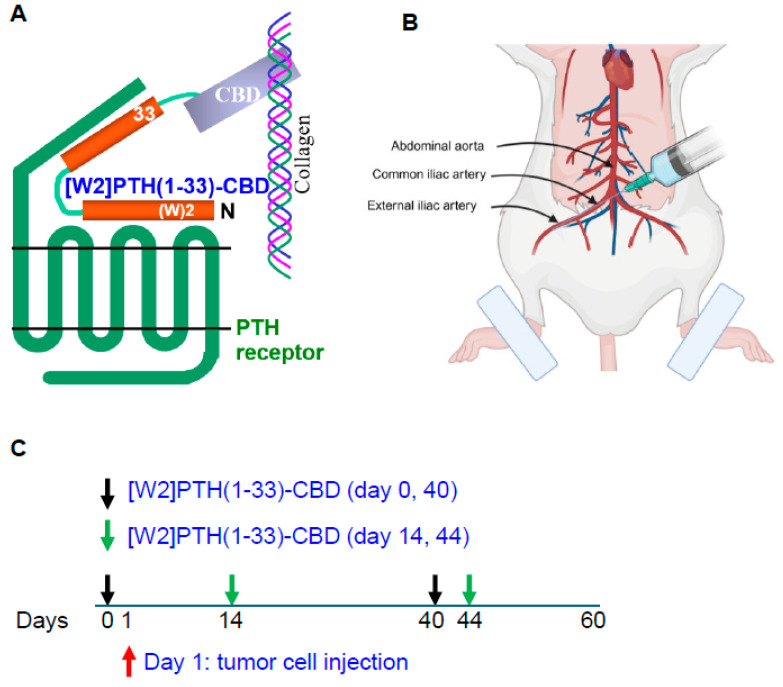
**Schematic representation of PTH antagonist, intra-iliac artery inoculation, and treatment regimen in mice.** (**A**) Interaction of [W2]PTH(1-33)-CBD with the PTH receptor and collagen type I in bone. (**B**) A schematic diagram illustrates intra-iliac artery inoculation of cancer cells in a mouse cartoon. (**C**) Early and delayed treatment regimen model. [W2]PTH(1-33)-CBD was given on days 0 and 40 after tumor cell inoculation (Early treatment, black arrows) and on days 14 and 44 for treatment design (Delayed treatment, green arrows).

**Figure 5:**
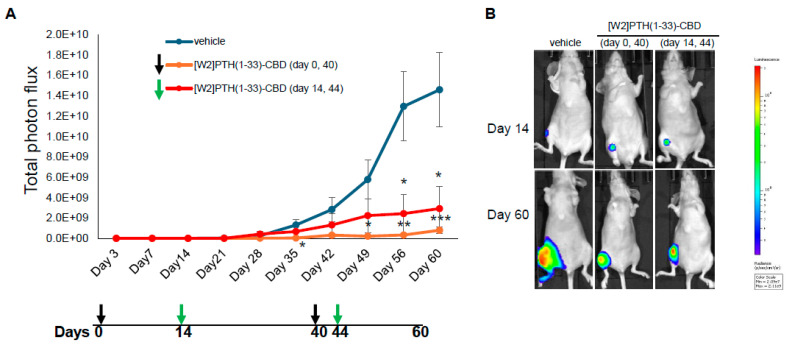
**Inhibition of breast tumor growth in bone by [W2]PTH 1-33 CBD: Intra-iliac artery injection of tumor cells.** Nude mice n = 5/group were administered vehicle (group 1) or 1000 µg/kg on days 0 and 40 (group 2), and Group 3 was administered [W2]PTH(1-33)-CBD 1000 µg/kg on days 14 and 44. On day 1, MDA-MB-231-BM/luc+ cells (2.5 × 10^5^) were injected into the iliac artery to selectively seed cancer cells into the hind limb bones of mice. (**A**) MDA-MB-231-BM/luc+ tumor growth in the hindlimb was quantitated by bioluminescence. (**B**) Representative BLI images taken on day 14 and day 60 were shown. BLSI is depicted using a color scale (right), where blue indicates low and red indicates high radiance levels (photons/sec/cm^2^/sr). All values shown are mean ± SEM. * *p* < 0.05, ** *p* < 0.01, *** *p* < 0.001 compared to vehicle.

**Figure 6:**
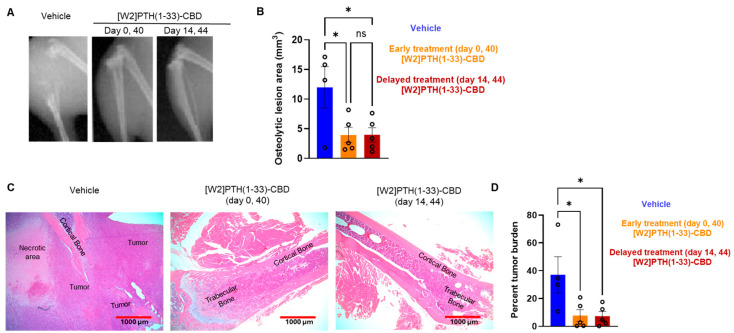
Radiographic and histological analysis revealed reduced osteolytic lesions by bone-targeted PTH antagonist [W2]PTH 1-33 CBD. (**A**) A Representative radiograph at day 60 of mice hindlimb from an intra-iliac experiment with three groups was shown. Group 1, vehicle; Group 2, [W2]PTH(1-33)-CBD was administered subcutaneously at a dose of 1000 µg/kg at day 0 and day 40 after tumor cell inoculation; Group 3, [W2]PTH(1-33)-CBD was administered subcutaneously at a dose of 1000 µg/kg at day 14 and day 44 after tumor cell inoculation. (**B**) The osteolytic lesion area was quantitated from X-ray images using image J. All values shown are mean ± SEM, n = 5 mice per group * *p* < 0.05, compared to vehicle; ns, not significant. (**C**) H&E images show that tumors occupy most of the area of the hind limb in the vehicle group, and [W2]PTH(1-33)-CBD treated protected bone robustly from tumor destruction. (**D**) The bar graph shows the percentage tumor burden in bone measured using ImageJ software. * *p* < 0.05.

Direct, intra-tibial inoculation of tumor cells into the marrow space has several drawbacks, including bypassing the early steps of metastasis, potential damage to local tissues from the procedure, and inconsistent tumor take in mice. To better model the early steps of metastasis, the hematogenous spread of cancer cells, and early-stage bone colonization, intra-iliac artery inoculation of tumor cells was utilized [16]. Two treatment strategies were compared: a early treatment regimen (drug administered 24 h tumor cell inoculation on days 0 and 40); and a treatment regimen (drug administered 14 and 44 days tumor establishment in bone) (Figure 4). For this experiment, [W2]PTH(1-33)-CBD was selected as it exhibited the highest overall drug efficacy both in vitro [13] and in vivo (Figure 5). The intra-iliac artery procedure resulted in more consistent tumor take and larger tumors that caused significant bone destruction by day 60 (Figure 5). Bioluminescent images of all mice from the vehicle, [W2]PTH(1-33)-CBD early treatment regimen and [W2]PTH(1-33)-CBD delayed treatment regimen at day 14 and day 60 were shown along with day 60 X-ray images in Supplementary Figure S4. Administration of [W2]PTH(1-33)-CBD at day 0 (early treatment regimen) significantly reduced tumor burden on days 35 and 49–60 (Figure 5A). Administration of [W2]PTH(1-33)-CBD on days 14 and 44 after tumor establishment (treatment regimen, red line (Figure 5A) significantly decreased tumor burden on days 56 and 60 (Figure 5A,B). Both drugs significantly attenuated tumor-induced osteolytic destruction (Figure 6A,B). These findings demonstrate that [W2]PTH(1-33)-CBD is effective in both early and delayed treatment settings to inhibit tumor growth and osteolytic destruction.

### 3.4. Histological Analysis of Intra-Iliac Tumors and Bone

Tibia from mice used in the intra-iliac experiments were prepared for H&E. The photomicrographs are shown at the diaphysis near the proximal tibial growth plate. However, these structures were only visible in the [W2]PTH(1-33)-CBD group. For the vehicle group, H&E sections were collected from the expected diaphyseal region, but only minimal bone tissue was detected (Figure 6C). In the vehicle group, most bony tissue is replaced by tumor, whereas the [W2]PTH(1-33)-CBD group clearly shows preservation of cortical and trabecular bone (Figure 6C).

## 4. Discussion

The present study used two mouse models for breast cancer bone metastasis (intra-tibial and intra-iliac injection of cancer cells) to demonstrate that a single subcutaneous injection of bone-targeted PTHrP antagonists effectively inhibits breast cancer bone metastases. The engineered antagonists, PTH(7-33)-CBD and [W2]PTH(1-33)-CBD, fuse a type 1 collagen binding domain to the antagonist peptide to enhance bone specificity, overcoming limitations observed in earlier PTH/PTHrP antagonists such as poor half-life and lack of targeted delivery [10,11]. PTH(7-33)-CBD and [W2]PTH(1-33)-CBD significantly reduced tumor burden and osteolytic bone destruction in preclinical models, and also induced apoptosis of breast cancer cells in vitro and inhibited cyclic AMP accumulation in osteosarcoma cells [13] comparable to the activity of the parent compound PTH(7-34). These findings indicate that addition of the CBD moiety does not compromise the biological function of the parent compound PTH(7-34) and align with accumulating evidence underscoring the pivotal role of PTHrP signaling in metastatic progression within the bone microenvironment.

A recent study reported that PTHrP inhibition reversed epithelial-to-mesenchymal transition (EMT) and diminished cancer stem cell populations in triple-negative breast cancer [19], further validating PTHrP as a critical driver of metastatic behavior. By directly interfering with the PTH/PTHrP axis, the bone-targeted antagonists in the present study could have disrupted tumor-stromal interactions essential for metastatic colonization and osteolysis. The bone-selective delivery of these compounds has been demonstrated by our prior work with a quantitative biodistribution analysis indicating that CBD-fused PTH antagonists localize primarily to bone with minimal soft tissue retention [13,20].

Intermittent administration of the PTH (1-34) agonist peptide has been shown to reduce tumor engraftment in bone metastasis models [4,21]. The anti-metastatic effects of the PTH(1-34) agonist was proposed to be primarily through stimulating bone formation and remodeling of the metastatic niche to inhibit cancer cell recruitment and reduce metastasis. PTH(1-34) was shown to modify VCAM-1 expression and preserve bone microarchitecture [4]. However, concerns about potential side effects, such as osteosarcoma, limit the clinical use of PTH(1-34) and other PTH agonist agents [22]. The mechanisms of therapeutic efficacy of PTH/PTHrP agonist drugs are fundamentally different from PTH(7-33)-CBD and [W2]PTH(1-33)-CBD. The latter antagonist drugs are targeted to type 1 collagen in the bone to concentrate the drug at the bone metastatic site, explicitly attenuating tumor-induced bone resorption without promoting broad anabolic activity, thereby offering potential advantages in mitigating skeletal complications associated with metastasis. Although in the intra-iliac study, one treatment group received [W2]PTH(1-33)-CBD one day prior to tumor cell inoculation. This was not considered a prevention treatment paradigm as the intra-tibial and intra-iliac injection models bypass the early steps of metastatic dissemination. Any potential preventive effect, therefore, remains speculative and would require additional studies in models that mimic the complete metastatic cascade from primary tumor dissemination to colonization in bone.

The findings from the present study provide strong evidence for the efficacy of bone-targeted PTH/PTHrP antagonists in preclinical models; however, we would also acknowledge some limitations that may impact their translational relevance and highlight areas for future investigation. A limitation of the present study is that all in vivo experiments were performed in immunodeficient mice to allow human tumor cell engraftment. This approach is well established for modeling bone metastases; however, it does not capture the potential contributions of the host immune system. Testing in immunocompetent mouse models will therefore be important for translational relevance, and such studies will be performed in the future to evaluate the efficacy of bone-targeted PTHrP antagonists in the context of an intact immune system. Although we used two distinct mouse models (tibial and iliac) to evaluate metastatic growth, these approaches did not include a non-metastatic control model. Incorporating such a model in future studies would help delineate metastasis-specific effects more clearly and provide additional insight. This will further refine our understanding of the selective impact of PTHrP antagonists on bone metastasis. Finally, the present work was designed as a proof-of-concept study and did not include comparator arms with approved therapies for advanced breast cancer, such as palbociclib or alpelisib [23,24]. Future comparative studies will be necessary to benchmark bone-targeted PTHrP antagonists against existing therapies and define their relative therapeutic potential.

In addition to MDA-MB-231 BM Luc+ cells, we previously demonstrated antagonist activity of PTH(7-33)-CBD and [W2]PTH(1-33)-CBD in SaOS-2 osteosarcoma cells, where [W2]PTH(1-33)-CBD showed superior efficacy in blocking PTHR1 signaling [13]. Importantly, the CBD-fused antagonist peptides exhibited no cytotoxicity in non-tumorigenic cell types, including MCF10A mammary epithelial cells, BJ5TA human fibroblasts, and THP-1 monocytes [13]. Toxicity studies conducted in mice revealed no observed toxicity [20]. Taken together, these findings suggest that these PTH/PTHrP antagonists fused with CBD may selectively target malignant cells in the bone microenvironment while sparing normal tissues.

The results of this study underscore the potential of targeted PTHrP antagonists in managing bone metastases from breast cancer. By effectively blocking PTHR1 signaling, these antagonists inhibit osteoclast activation and tumor progression [13], addressing a critical limitation of current treatments. Future investigations should prioritize pharmacokinetic optimization, efficacy assessment across diverse metastatic models, and immunogenicity evaluation with prolonged administration. Such efforts will be instrumental in advancing these compounds toward clinical translation.

## 5. Conclusions

In summary, this study provides compelling preclinical evidence supporting the therapeutic potential of bone-targeted PTHrP antagonists, PTH(7-33)-CBD and [W2]PTH(1-33)-CBD, in inhibiting breast cancer bone metastases. By combining receptor antagonism with bone-targeted delivery, these agents selectively disrupt the interaction between cancer cells and bone, mitigating osteolytic damage and thereby avoiding the risks associated with anabolic PTH agonists. These findings lay a strong foundation for further development and clinical translation of targeted PTHrP blockade as a novel strategy to manage skeletal metastases in breast cancer.

## Supplementary Materials

The following supporting information can be downloaded at: https://www.mdpi.com/article/10.3390/cancers17172933/s1, Figure S1. In vivo bioluminescence of the intratibial breast metastatic tumor growth on week 10 post-administration with the vehicle, PTH(7-34) control, PTH(7-33)-CBD, and [W2]PTH(1-33)-CBD following intra-tibial injections of MDA-MB-231-BM/luc+. BLSI is depicted using a color scale (right), where blue indicates low and red indicates high radiance levels (photons/sec/cm^2^/sr); Figure S2. In vivo radiographic images of mice bearing bone breast tumor on week 10 post-administration of vehicle, PTH(7-34) control, PTH(7-33)-CBD, and [W2]PTH(1-33)-CBD following intra-tibial injections of MDA-MB-231-BM/luc+; Figure S3. Ex vivo micro-CT images of mice (n = 5–7) tibia 10 weeks after administration with the vehicle, PTH(7-34) control, PTH(7-33)-CBD, and [W2]PTH(1-33)-CBD subcutaneously one dose of 1000 ug/kg following intratibial injections of MDA-MB-231-BM/luc+; Figure S4. Following the intra-iliac injection of tumor cells (MDA-MB-231-BM/luc+), the nude mice (n = 5/group) were administered with vehicle and [W2]PTH(1-33)-CBD 1000 μg/kg subcutaneously on days 0 and 40. Another group of mice was administered the same drug on days 14 and 44. In vivo, bioluminescence imaging was performed on days 14 and 60 to assess tumor progression. Bioluminescent signal intensity is depicted using a color scale (right), where blue indicates low and red indicates high radiance levels (photons/sec/cm^2^/sr). Radiographic (X-ray) images on Day 60 were used to evaluate bone integrity and tumor-induced bone damage.
