# Supplementary material for: Bone Targeted Parathyroid Hormone Antagonists for Prevention of Breast Cancer Bone Metastases
Source: Cancers (Basel). 2025 Sep 8;17(17):2933. doi: 10.3390/cancers17172933 (PMC12427927; doi:10.3390/cancers17172933)
Supplement: Supplementary file 1 [file cancers-17-02933-s001.zip › cancers-3763480-supplementary.pdf]

cancers

MDPI

Article

Bone Targeted Parathyroid Hormone Antagonists for Prevention of Breast Cancer Bone Metastases

Muralidharan Anbalagan [1,†], Tulasi Ponnapakkam [1,2,†], Binghao Zou [1], Jarvis Williams [1], Fouad Saeg [1], Matthew E. Burow [3], Robert C. Gensure [4] and Brian G. Rowan [1,*]

1 Department of Structural and Cellular Biology, Tulane University School of Medicine, New Orleans, LA 70112, USA; manbalag@tulane.edu (M.A.); tponnapakkam@gmail.com (T.P.); bzou1@tulane.edu (B.Z.); jwilliams15@tulane.edu (J.W.); fsaeg@tulane.edu (F.S.)
2 Department of Chemistry, Xavier University of Louisiana, New Orleans, LA 70125, USA
3 Section of Hematology & Medical Oncology, Department of Medicine, Tulane University School of Medicine, New Orleans, LA 70112, USA; mburow@tulane.edu
4 Dartmouth Health Children's, Geisel School of Medicine, Lebanon, NH 03756, USA; robert.c.gensure@hitchcock.org
* Correspondence: browan@tulane.edu; Tel.: +1-504-988-1365
† These authors contributed equally to this work.

Supplementary figure legend

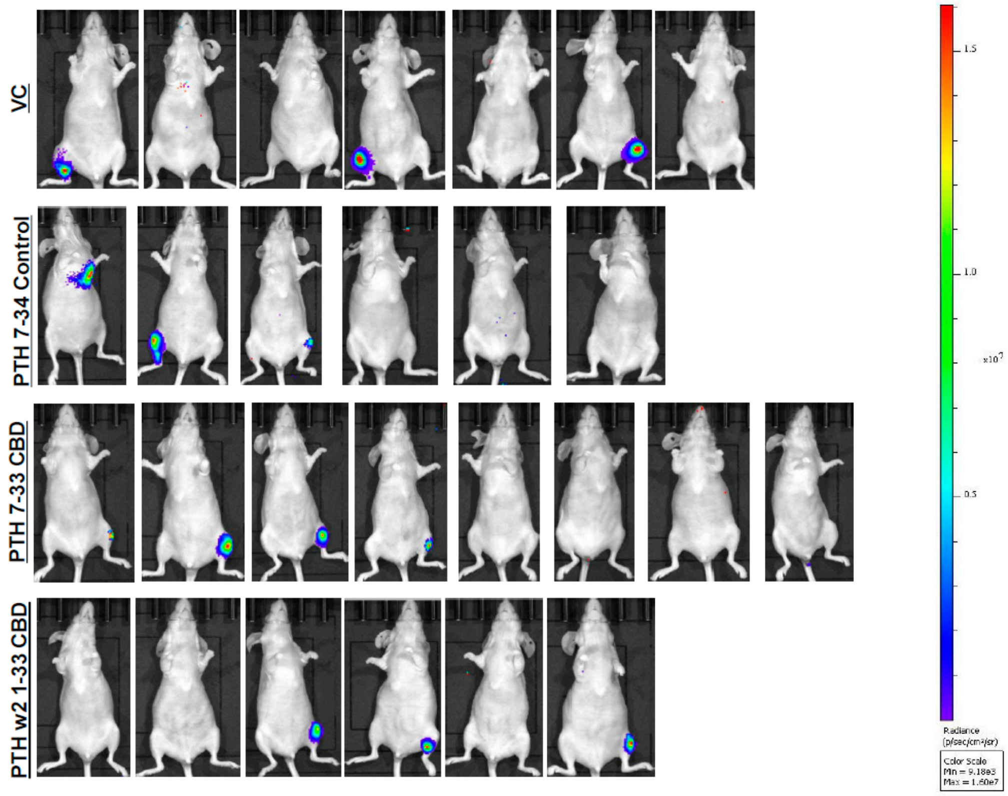

Figure S1. *In vivo* bioluminescence of the intratibial breast metastatic tumor growth on week 10 post-administration with the vehicle, PTH(7-34) control, PTH(7-33)-CBD, and [W2]PTH(1-33)-CBD following intra-tibial injections of MDA-MB-231-BM/luc+. BLSI is depicted using a color scale (right), where blue indicates low and red indicates high radiance levels (photons/sec/cm²/sr).

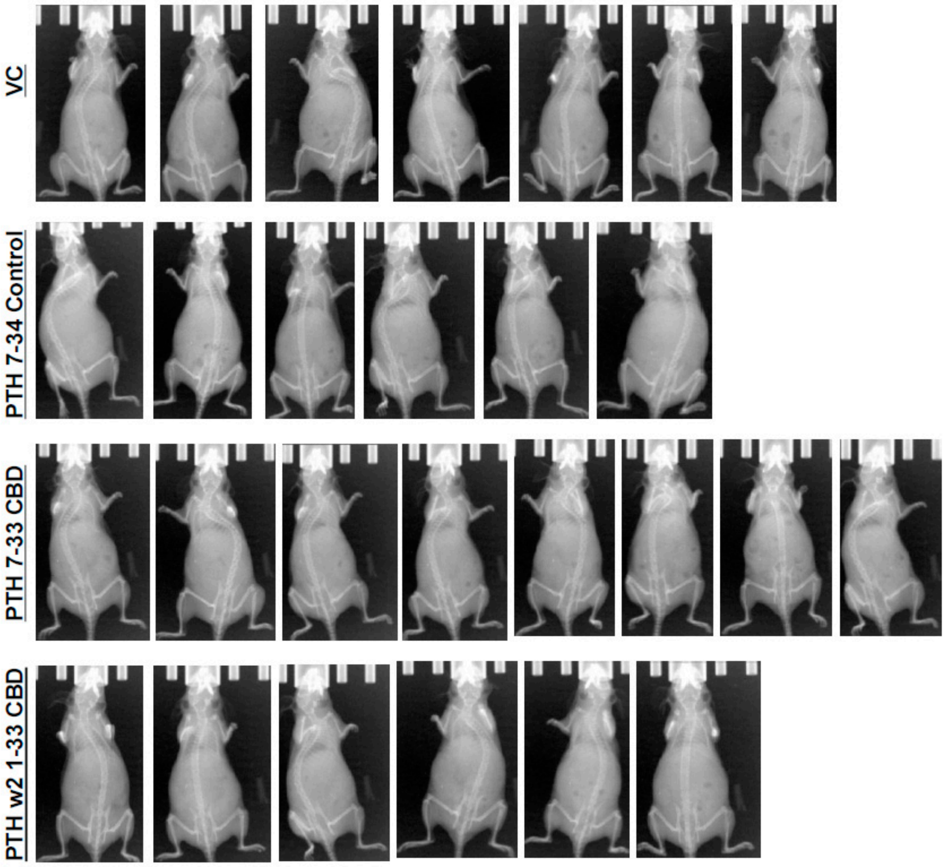

Figure S2. *In vivo* radiographic images of mice bearing bone breast tumor on week 10 post-administration of vehicle, PTH(7-34) control, PTH(7-33)-CBD, and [W2]PTH(1-33)-CBD following intratibial injections of MDA-MB-231-BM/luc+.

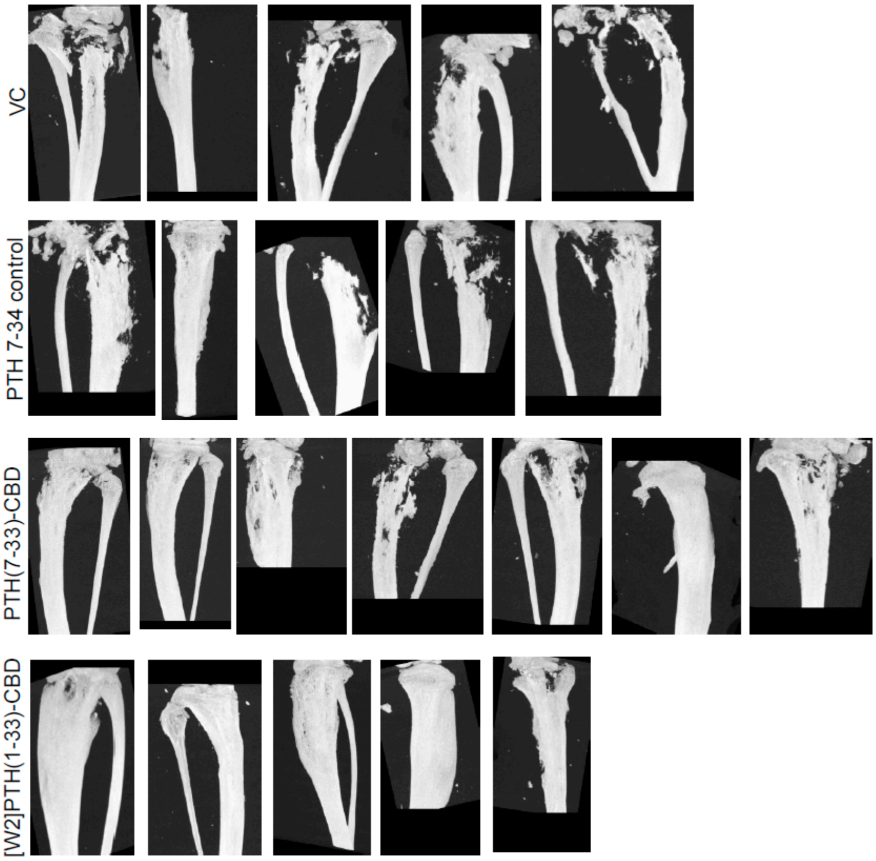

Figure S3. *Ex vivo* micro-CT images of mice (n = 5–7) tibia 10 weeks after administration with the vehicle, PTH(7-34) control, PTH(7-33)-CBD, and [W2]PTH(1-33)-CBD subcutaneously one dose of 1000ug/kg following intratibial injections of MDA-MB-231-BM/luc+.

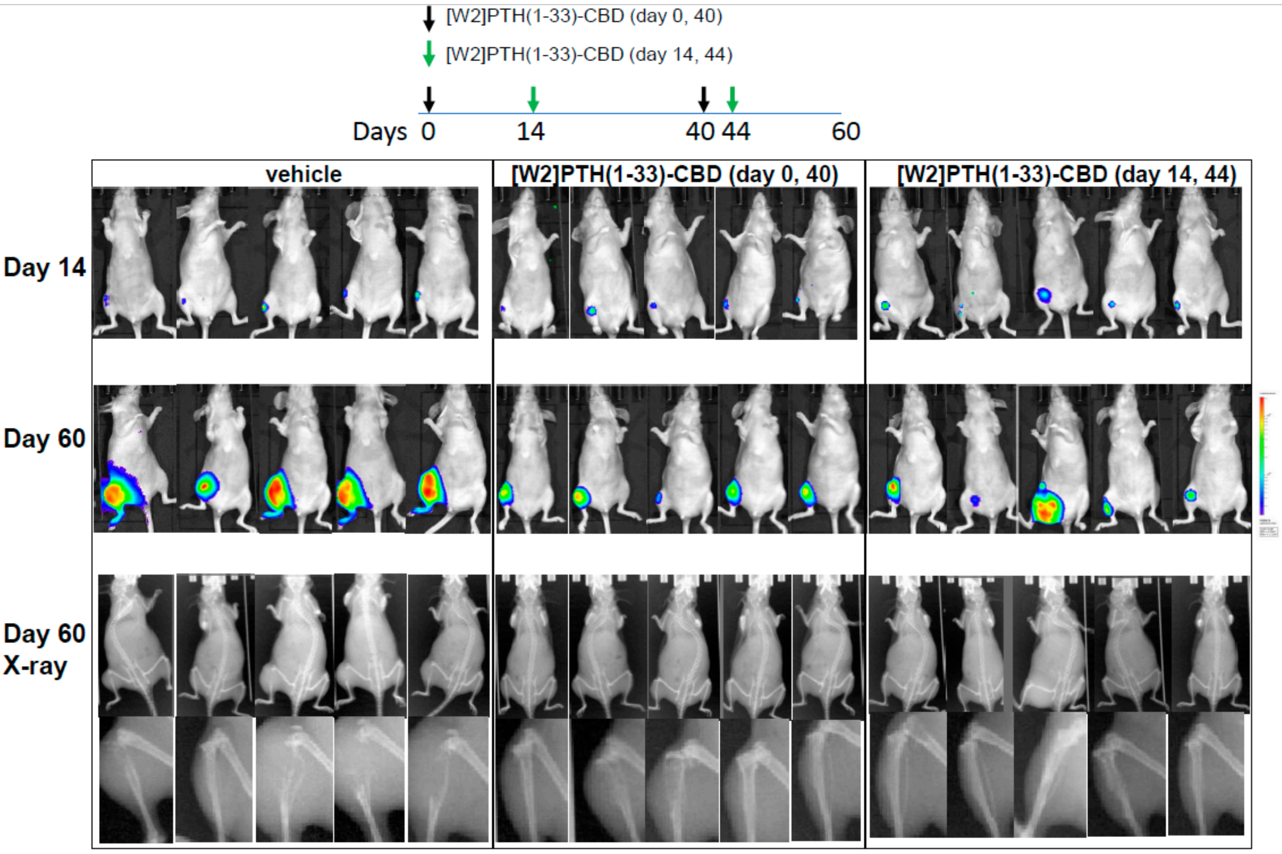

Figure S4. Following the intra-iliac injection of tumor cells (MDA-MB-231-BM/luc+), the nude mice (n=5/group) were administered with vehicle and [W2]PTH(1-33)-CBD 1000ug/kg subcutaneously on days 0 and 40. Another group of mice was administered the same drug on days 14 and 44. *In vivo,* bioluminescence imaging was performed on days 14 and 60 to assess tumor progression. Bioluminescent signal intensity is depicted using a color scale (right), where blue indicates low and red indicates high radiance levels (photons/sec/cm²/sr). Radiographic (X-ray) images on Day 60 were used to evaluate bone integrity and tumor-induced bone damage.
